# Mediation role of interpersonal problems between insecure attachment and eating disorder psychopathology

**DOI:** 10.1007/s40519-024-01673-5

**Published:** 2024-06-21

**Authors:** Marco Carfagno, Eugenia Barone, Eleonora Arsenio, Rosaria Bello, Luigi Marone, Antonio Volpicelli, Giammarco Cascino, Alessio Maria Monteleone

**Affiliations:** 1 Department of Psychiatry, University of Campania “Luigi Vanvitelli”, Naples, Italy; 2 Department of Medicine, Surgery and Dentistry ‘Scuola Medica Salernitana’, Section of Neurosciences, University of Salerno, Salerno, Italy

**Keywords:** Eating disorders, Attachment, Interpersonal problems, Psychopathology, Mediation analysis

## Abstract

**Purpose:**

Although insecure attachment and interpersonal problems have been acknowledged as risk and maintaining factors of eating disorders (EDs), the mediating role of interpersonal problems between attachment style and ED psychopathology has been poorly explored. The purpose of this study was to investigate the mediating role of interpersonal problems between insecure attachment and ED psychopathology.

**Methods:**

One-hundred-nine women with anorexia nervosa and 157 women with bulimia nervosa filled in the Eating Disorder Inventory-2 (EDI-2) and the Experiences in Close Relationships (ECR) revised scale to assess ED core symptoms and attachment styles, respectively. Interpersonal difficulties were evaluated by the Inventory of Interpersonal Problems (IIP-32). A mediator’s path model was conducted with anxious and avoidant attachment subscores as independent variables, ED core symptoms as dependent variables and interpersonal difficulties as mediators. The diagnosis was entered in the model as a confounding factor.

**Results:**

The socially inhibited/avoidant interpersonal dimension was a mediator between avoidant attachment and the drive to thinness as well as between avoidant attachment and body dissatisfaction. An indirect connection was found between attachment-related anxiety and bulimic symptoms through the mediation of intrusive/needy score.

**Conclusions:**

Social avoidance and intrusiveness mediate the relationships between avoidant and anxious attachment styles and ED psychopathology. These interpersonal problems may represent specific targets for psychotherapeutic treatments in individuals with EDs and insecure attachment.

**Level of evidence:**

Level III: Evidence obtained from well-designed cohort or case–control analytic studies.

## Introduction

The attachment theory states that an innate predisposed psychobiological system motivates people to seek proximity to significant others in times of distress [1]. Repeated interactions between the infant and attachment figures, who demonstrate to be available, sensitive and responsive to the infant’s needs, will lead the individual to develop an attachment security and build positive mental representations of self and others. In contrast, when caregivers are not available or are unpredictable negative “internal working models” of self and others are built [2, 3]. These cognitive-affective schema of relational expectations, emotions, and behaviour that results from early interactions with attachment figures persist in adulthood and are named attachment styles [4, 5]. Secure attached individuals can deal with a variety of emotions experienced in interpersonal interactions, to communicate and accept their feelings, to seek aid and proximity when feeling overwhelmed, to reflect on their own mental states and on those of the others (i.e., mentalizing abilities) [6, 7]. Insecure attachment includes two main categories: the anxious or preoccupied and the avoidant or dismissive style. A caregiver who facilitates the formation of an anxious attachment in offspring typically exhibits inconsistent and unpredictable reactions, excessive protectiveness and criticism, emotional volatility, anxiety, and emotional reliance on the child. Indeed, attachment anxiety is related to a person who worries that a significant other will not be available and responsive in times of difficulty [8]. On the other hand, a parent who is inclined towards developing an avoidant attachment in offspring typically displays emotional detachment and a lack of responsiveness to the child's requirements, advocates for premature self-reliance, and downplays or overlooks emotional displays. Thereby attachment avoidance is related to a negative view of the others with a trend to self-rely, to maintain behavioural independence and to minimize emotion expression [9]. To address dysfunctional beliefs and emotions arising from insecure attachment styles, attachment-based psychotherapy was developed [10]. This form of psychoanalytic psychotherapy has been effective in the treatment of various psychiatric disorders, including anxiety and depression [10].

Insecure attachment is a risk factor for eating disorders (EDs) [8, 11, 12]. Indeed, insecure attachment was associated to dysfunctional eating behaviours in the general population [13], and people with EDs showed a higher prevalence of insecure attachment as compared to healthy individuals [14]. A systematic review [15] also quantified the association between insecure attachment and EDs showing a medium to high effect size (r = 0.41; d = 1.31). Attachment insecurity has been associated with the severity of ED-related symptoms and proved to be a transdiagnostic risk factor for EDs [8, 16]. In addition, attachment insecurity has a negative effect on psychotherapy outcomes as shown by heightened dropping out in patients with higher attachment avoidance and worse treatment outcomes in patients with higher attachment anxiety [17, 18].

Several psychological and biological mechanisms have been proposed to explain the association between insecure attachment and ED psychopathology. In their meta-analytic review Cortes-Garcia et al. [19] found that emotion dysregulation and depressive symptoms were the strongest mediators of such a relationship. Neuroticism, perfectionism, problems with mindfulness and social comparison were also observed as mediators but with a low to moderate effect [19]. Subsequent studies suggested further mediators such as lack of confidence towards body feelings and lack of emotional acceptance [20]. This is in line with the four mechanisms proposed by Faber et al. [13] to explain the association between insecure attachment and ED psychopathology in a non-clinical population: a direct and general vulnerability, difficulties with emotions, negative self-esteem, and interpersonal problems. Insecure attached people have poor conflict management and difficulties in sharing intimacy [21] and show loneliness and decreased relationship satisfaction [22, 23]. In individuals with EDs insecure attachment was associated with heightened emotional and biological vulnerability to an acute social challenge [24]. However, the relationship between insecure attachment styles and interpersonal difficulties as well as the mechanisms by which interpersonal difficulties make individuals with insecure attachment more vulnerable towards ED psychopathology have been under investigated. This is an important literature gap also considering the centrality of interpersonal difficulties in the psychopathology of EDs [15, 25]. As a proof of this, patients recalled social difficulties as preceding their ED and worsening after illness onset [26]. Experimental studies have demonstrated heightened sensitivity to social stress [27, 28] and attentional bias towards social rejection [29] in individuals with EDs and interpersonal difficulties have been shown to predict treatment outcome [30].

The aim of this study was to explore which specific interpersonal problems (i.e., submissiveness, assertiveness, hostility, intrusiveness, having difficulties supporting others, being excessively caring or dependent) mediate the relationship between insecure attachment and ED psychopathology. Given that avoidant people are usually cold, introverted, and competitive, while anxious individuals are often overly emotional [31, 32], we hypothesized that hostility and having difficulties supporting others were involved in the association between avoidant attachment and ED psychopathology, while being excessively caring or dependent was mediating between the anxious style and ED psychopathology.

## Methods

### Participants

A consecutive sample of individuals attending the ED outpatient centre at University of Campania L. Vanvitelli was invited to take part into the study if the following criteria were met: (a) current diagnosis of anorexia nervosa (AN), atypical AN or bulimia nervosa (BN) according to DSM-5; (b) age ≥ 14 years; (c) absence of comorbid bipolar disorder, schizophrenia or substance-related disorder. All the participants gave their written consent after being thoroughly informed about the nature of the study. Socio-demographic and clinical data were collected through a semi-structured interview conducted by an expert psychiatrist. Diagnosis and comorbidities were assessed at admission to the ED unit by a trained psychiatrist and then confirmed through the Structured Clinical Interview for DSM-5 Disorders–Research Version [33]. Each participant was asked to fill in self-report questionnaires as part of the centre’s clinical routine before entering treatment program. The study was approved by University of Campania L. Vanvitelli ethical committee (protocol number 09.32-20210013912).

### Clinical questionnaires

- ED-related symptoms and psychopathology were measured through the Eating Disorders Inventory-2 (EDI-2) [34, 35], which is a self-administered questionnaire encompassing 11 subscales: impulsivity, fear, body dissatisfaction, social insecurity, asceticism, perfectionism, interpersonal distrust, ineffectiveness, drive to thinness, interoceptive awareness, bulimia, and maturity. The scores of core ED symptoms (namely, bulimia (Cronbach’s α = 0.87), body dissatisfaction (Cronbach’s α = 0.84) and drive to thinness (Cronbach’s α = 0.91) subscales) were included in the analyses.
- The Experiences in Close Relationships (ECR) revised scale is a self-reported questionnaire evaluating the attachment styles [36, 37], in a romantic relationship, which represents an indirect measure of early interactions with caregivers [38]. The ECR provides two main subscales measuring the anxious insecure attachment (Cronbach’s α = 0.82) and the avoidant insecure attachment (Cronbach’s α = 0.88).
- The Inventory of Interpersonal Problems 32-item version (IIP-32) [39] was employed to assess interpersonal functioning. This questionnaire explores interpersonal difficulties [39], which are conceptualized in terms of dominance and affiliation. These interpersonal features are divided into eight sub-scales: Overly Accommodating/Exploitable (Cronbach’s α = 0.73), Vindictive/ Self-centred (Cronbach’s α = 0.72), Intrusive/Needy (Cronbach’s α = 0.81), Socially Inhibited/Avoidant (Cronbach’s α = 0.83), Non-assertive (Cronbach’s α = 0.80), Cold/ Distant (Cronbach’s α = 0.75), Self-sacrificing/Overly nurturant (Cronbach’s α = 0.77), and Domineering/Controlling (Cronbach’s α = 0.79).

### Statistical analyses

The normality of data was tested through the Shapiro–Wilk test. Since most of the data were not normally distributed, the non-parametric Mann–Whitney *U* test was conducted to compare clinical differences between the AN group and the BN group. The Bonferroni correction was applied so that the level of significance was set at *p* < 0.0026. Mediation analyses were conducted by using JASP software [40]. A multiple mediator’s path model was run with attachment subscores as independent variables, ED core symptoms as dependent variables and interpersonal difficulties as mediators. The diagnosis was entered in the model as a confounding factor. The statistical significances of the mediating and indirect effects were assessed using bootstrapped procedure (namely, running percentile-based confidence interval of 5000 bootstrap) [41] and the maximum likelihood (ML) method [42].

## Results

### Sample characteristics

Participants’ mean age was 25.4 years (SD = 9.4, min = 14, max = 54). One-hundred-nine women with AN and 157 women with BN were recruited. Fifty-three (19.9%) patients reported a current diagnosis of an anxiety disorder, thirty-six (13.5%) reported a diagnosis of major depression. As seen in Table 1, differences between AN and BN emerged in terms of age, BMI, years of education, illness duration, and EDI-2 subscale for Bulimia (all *p* < 0.001). No significant differences emerged between individuals with AN and those with BN regarding insecure attachment styles and interpersonal issues except for intrusive/needy score (*p* = 0.007) and self-sacrifing/overly nurture score (*p* = 0.023) which did not persist after Bonferroni correction.

**Table 1:** Mann–Whitney *U* test comparing clinical differences between women with AN and BN

Parameters mean ± SD	AN women (109)	BN women (157)	U	*p*-value
Age	**22.889 ± 8.341**	**27.173 ± 9.810**	**5889.500**	**< 0.001**
BMI	**16.921 ± 2.205**	**22.034 ± 9.018**	**2365.500**	**< 0.001**
Age of onset	17.255 ± 4.709	17.973 ± 5.289	5270.000	0.242
Years of education	**12.000 ± 3.181**	**13.559 ± 3.381**	**2705.500**	**< 0.001**
Illness duration	**5.748 ± 6.929**	**9.449 ± 8.898**	**5270.500**	**< 0.001**
Eating Disorder Inventory 2 Body Dissatisfaction	14.440 ± 7.706	16.420 ± 7.243	7336.000	0.048
Eating Disorder Inventory2 Drive to Thinness	13.761 ± 6.872	16.197 ± 5.493	6816.500	0.005
Eating Disorder Inventory2 Bulimia	**1.963 ± 3.058**	**9.783 ± 6.163**	**2275.000**	**< 0.001**
Inventory of Interpersonal Problems 32 item Overly Accommodating/Exploitable	62.901 ± 12.654	66.236 ± 11.744	3339.000	0.099
Inventory of Interpersonal Problems 32 item Vindictive/ Self-centred	55.704 ± 12.193	56.300 ± 11.972	3778.000	0.711
Inventory of Interpersonal Problems 32 item Intrusive/Needy	55.732 ± 12.732	62.309 ± 16.039	2980.500	0.007
Inventory of Interpersonal Problems 32 item Socially Inhibited/Avoidant	66.859 ± 14.065	64.818 ± 13.039	4248.000	0.318
Inventory of Interpersonal Problems 32 item Non-assertive	60.704 ± 11.599	62.582 ± 10.873	3660.000	0.476
Inventory of Interpersonal Problems 32 item Cold/ Distant	59.930 ± 12.628	60.527 ± 12.157	3772.000	0.699
Inventory of Interpersonal Problems 32 item Self-sacrificing/Overly nurturant	61.620 ± 11.140	65.736 ± 10.832	3122.500	0.023
Inventory of Interpersonal Problems 32 item Domineering/Controlling	60.676 ± 16.321	64.527 ± 15.671	3314.500	0.085
Experience in Close Relationship Attachment anxiety	80.032 ± 22.685	80.507 ± 22.762	6693.500	0.997
Experience in Close Relationship Attachment avoidance	71.204 ± 23.239	68.563 ± 23.452	7201.000	0.328

*AN* anorexia nervosa, *BMI* Body Mass Index, *BN* bulimia nervosa, *SD* standard deviation, *U* Mann–Whitney U statistic. Bold data are significant data ( *p* < 0.0026 after Bonferroni correction)

### Mediation analysis

**Fig. 1:**
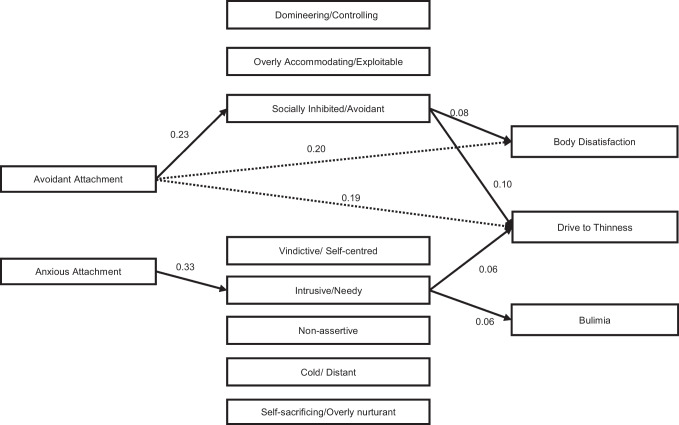
Path mediation model showing the association between insecure attachment, interpersonal problems and specific eating symptoms in women with eating disorders. Dotted lines represent significant direct effects, continuous lines represent significant indirect effects. The standardized coefficients are shown in the figure

Figure 1 shows the mediation model. A significant direct path between avoidant attachment, drive to thinness (b = 0.19, 95% CI = 0.04–0.34, *p* = 0.007) and body dissatisfaction (b = 0.20, 95% CI = 0.061–0.326, *p* = 0.004) emerged, displaying that higher attachment avoidance score was related to heightened drive to thinness and body dissatisfaction. Socially Inhibited/Avoidant score mediated both the associations between avoidant attachment and drive to thinness (b = 0.10, 95% CI = 0.036–0.204, *p* = 0.008) and between avoidant attachment and body dissatisfaction (b = 0.08, 95% CI = 0.027–0.172, *p* = 0.012). A significant indirect association emerged between attachment-related anxiety and bulimic symptoms through Intrusive/Needy score (b = 0.06, 95% CI = 0.01–0.04, *p* = 0.026). Intrusive/Needy also slightly mediated the association between anxious attachment and drive to thinness (b = 0.06, 95% CI = 0.004–0.142, *p* = 0.086). The diagnosis had a significant effect on Intrusive/Needy pointing that BN diagnosis may account for the indirect effect of attachment-related anxiety on bulimia through Intrusive/Needy score.

## Discussion

The aim of this study was to explore the mediation role of interpersonal difficulties between insecure attachment and eating-related core symptoms in a sample with AN and BN. Social inhibition/avoidance mediated the association of avoidant attachment with drive to thinness and body dissatisfaction, while intrusiveness mediated between anxious attachment and bulimic symptoms.

No difference has been observed in the present sample among individuals diagnosed with AN and BN in relation to ED psychopathology, insecure attachment style, and interpersonal difficulties. This is consistent with previous literature: although the relationship between insecure attachment and ED psychopathology has been widely acknowledged [8, 17], no clear association between ED diagnoses and insecure attachment dimensions has been previously detected [8, 17]. Likewise, avoidance of expressing feelings to others giving priority to other people’s feeling over their own and interpersonal distrust and conflict with others were described as the interpersonal difficulties differentiating individuals with AN and those with BN [43], while a few studies have assessed interpersonal difficulties in terms of dominance and affiliation and failed to identify differences between AN and BN [44, 45].

We hypothesized that hostility and being cold were involved in the association between avoidant attachment and ED psychopathology, while being excessively dependent was mediating between the anxious style and ED psychopathology. The present findings align with our hypotheses given that social avoidance is a component of the “cold” dimension and has been described as highly associated with the cold/distant subscore [45, 46], while intrusiveness is included in the “dependent” dimension [39]. Individuals with avoidant attachment styles use to distance themselves from forming close connections, thereby safeguarding themselves from potential rejection [31, 47]. Conversely, individuals with anxious attachment styles are characterized by difficulties respecting personal space and seeking excessive reassurance or attention from others. Therefore, these findings align with attachment theory [31], indicating that avoidance of social interactions may make individuals with avoidant attachment style more vulnerable to body dissatisfaction and drive to thinness, while the need of avoiding rejection to foster intimacy, even at the expense of respecting others’ personal space and autonomy, may promote bulimic symptoms in those with higher anxious attachment styles. The latter finding may be suggested above all in individuals with BN given the significant effect of BN diagnosis on intrusive/needy score observed in the mediation model. Considering literature on the association between interpersonal difficulties and ED psychopathology [43, 48], the suggested role of social exposure avoidance has been supported in individuals with EDs and avoidant attachment, while intrusiveness has emerged as an under-detected interpersonal problem with relevance in individuals with EDs and anxious attachment. In addition, it is possible to suggest that eating-related symptoms may represent dysfunctional strategies to cope with the highlighted interpersonal difficulties encountered by individuals with insecure attachment. Eating symptoms may allow patients to divert their attention from their undesired feelings in the context of social exposure or perception of abandonment. The current findings corroborate a wide body of research outlining that an insecure attachment style fosters ED symptomatology [8, 31]. However, previous studies have gathered evidence about the mediating role of emotion regulation difficulties [19, 20] and self-esteem [13]. Although interpersonal difficulties were hypothesized to mediate this association [11, 13], social comparison and behavioural inhibition related to sensitivity to punishment have been the only investigated interpersonal dimensions so far [49, 50]. To the best of our knowledge, this study represents the first attempt to investigate the mediating role of interpersonal problems, measured by the IIP-32 questionnaire, between insecure attachment and ED symptoms.

Previous research has consistently shown a significant association between high insecure attachment and more severe ED symptoms, as well as unfavourable treatment outcomes, across all subtypes of EDs [51, 52]. In addition, improvements in attachment anxiety and avoidance have been associated with reduced interpersonal difficulties [53]. Therefore, these findings underscore the importance of interventions aimed at improving attachment functioning to mitigate the severity of ED symptoms. In this line, evidence-based psychotherapies (i.e., the Focal Psychodynamic Therapy [54]) targeting attachment-related issues may be suggested for individuals with EDs and high insecure attachment [55]. Furthermore, specific interpersonal problems such as social avoidance and lack of interpersonal boundary emerged in this study as mediating between insecure attachment and ED symptoms. Therefore, interventions proved to ameliorate interpersonal problems in EDs may be suggested to address these interpersonal difficulties considering their relationships with attachment experiences. In this line, social inhibition can be effectively addressed using cognitive-behavioral therapy, which reduces social anxiety and improves social functioning by targeting negative thought patterns and avoidance behaviours [56]. For those exhibiting high levels of intrusiveness, interpersonal psychotherapy may be particularly effective, as it improves communication skills and management of interpersonal disputes, thereby mitigating the effects of intrusiveness in relationships [57].

Concluding, attachment styles and their relationships with difficulty approaching others and imposing one’s needs with difficulty respecting the personal boundaries of other people may represent more tailored treatment targets in individuals with EDs and high avoidant and anxious attachment dimensions, respectively.

### Strength and limits

The main strength of the study is that it is the first to examine interpersonal styles in terms of dominance and affiliation as mediators of the relationship between insecure attachment and ED psychopathology. Limitations of the study need to be acknowledged. First, a cross-sectional approach was employed and precluded to draw causality. A further consequence of this issue is that, although the ECR questionnaire is considered a reliable indicator of infant attachment experiences [27], its scores may reflect the cognitive-affective schema employed in current peer relationships rather than a trait. Second, the use of self-report questionnaires may induce bias in the reported findings, although there is large agreement in considering self-report attachment instruments appropriate [41]. Third, the inclusion of participants into a unique sample prevents assessing differences between ED diagnoses. However, literature data do not support specific associations between ED diagnoses and attachment insecure styles [13] and the diagnosis factor was considering in the model suggesting a higher relevance of the anxious attachment pathway in individuals with BN. Fourth, the lack of a matched healthy control sample does not allow to verify if the identified mediation effects specifically characterize individuals with EDs.

### What is already known on this subject?

Insecure attachment style fosters ED symptomatology and previous studies have gathered evidence about the mediating role of emotion regulation difficulties and self-esteem. Although interpersonal difficulties were hypothesized to mediate this association, social comparison and behavioural inhibition related to sensitivity to punishment have been the only investigated interpersonal dimensions so far. To the best of our knowledge, this study represents the first attempt to investigate the mediating role of interpersonal problems, measured by the IIP-32 questionnaire, between insecure attachment and ED symptoms.

### What this study adds?

Our findings suggest that interpersonal problems, namely social avoidance and intrusiveness, mediate the relationship between avoidant and anxious attachment styles and ED psychopathology, emphasizing the importance of addressing specific interpersonal difficulties in psychotherapeutic interventions for individuals with EDs and insecure attachment.

## Data Availability

The datasets generated during and/or analysed during the current study are available from the corresponding author on reasonable request.
